# Efficacy of Chemotherapy in Survival of Stage I Nasopharyngeal Carcinoma

**DOI:** 10.3389/fonc.2021.735817

**Published:** 2021-10-14

**Authors:** Jia-Lin Ma, Shi-Ting Huang, Yan-Ming Jiang, Xin-Bin Pan

**Affiliations:** Department of Radiation Oncology, Guangxi Medical University Cancer Hospital, Nanning, China

**Keywords:** nasopharyngeal carcinoma, stage I, chemotherapy, survival, prognosis

## Abstract

**Purpose:**

To identify whether chemoradiotherapy improves survival of stage I nasopharyngeal carcinoma (NPC).

**Materials and Methods:**

NPC patients were extracted from the Surveillance, Epidemiology, and End Results database between 2010 and 2015. Pathologically confirmed stage T1N0M0 (the 7^th^ edition AJCC) were investigated. Overall survival (OS) and cancer-specific survival (CSS) were compared between the radiotherapy and chemoradiotherapy groups using the Kaplan-Meier method and propensity score matching (PSM) analyses.

**Results:**

This study included 91 (40.27%) patients in the chemoradiotherapy group and 135 (59.73%) patients in the radiotherapy group. Before PSM, chemoradiotherapy was associated with worse 3-year OS (74.31 *vs* 87.23%; *P* = 0.025) and 5-year OS (64.28 *vs* 83.12%; *P* = 0.001) compared to those associated with radiotherapy. Similarly, chemoradiotherapy showed worse 3-year CSS (87.01 *vs* 96.97%; *P* = 0.028) and 5-year CSS (80.39 *vs.* 96.97%; *P* = 0.002) than those of radiotherapy. After PSM, chemoradiotherapy revealed worse 5-year OS (63.10 *vs.* 82.49%; *P* = 0.031) and CSS (80.95 *vs.* 93.70%; *P* = 0.016) than radiotherapy. The multivariate regression analysis revealed that chemoradiotherapy was an independent risk prognostic factor for OS and CSS before and after PSM.

**Conclusion:**

Radiotherapy alone is recommended for stage I NPC patients.

## Introduction

Chemoradiotherapy is recommended for stage II and locoregionally advanced nasopharyngeal carcinoma (NPC) (1–4). In contrast, radiotherapy alone provides satisfactory treatment outcomes for stage I NPC. The 5-year overall survival (OS) is higher than 95.0% (5–8). However, about 5.0% patients would suffer from local-regional recurrence or distant metastasis in the first 5 years (5, 8, 9). Whether addition of chemotherapy to radiotherapy improves survival for stage I NPC patients is not well investigated. This study was conducted to assess the efficacy of chemotherapy in survival of stage I NPC. The results of this study might help clinicians to make a better treatment option for this subgroup patients.

## Materials and Methods

### Patients

NPC patients were extracted from the Surveillance, Epidemiology, and End Results (SEER) database from 2010 to 2015. The inclusion criteria were as follows: (1) pathologically confirmed NPC; (2) patients with definite TNM stages of the 7^th^ edition American Joint Committee on Cancer (AJCC); (3) patients with stage I (T1N0M0); and (4) patients who received radiotherapy. Patient characteristics, including age, sex, race, tumor grade, pathology, and chemotherapy, were extracted.

### Treatments and Endpoints

The included patients were divided into chemoradiotherapy and radiotherapy groups. Patients in the chemoradiotherapy group received radiotherapy combined with chemotherapy. Patients in the radiotherapy group received radiotherapy alone.

OS was the primary endpoint, which was defined as the time until death due to any cause in the SEER database. Cancer-specific survival (CSS) was the secondary endpoint, which was defined as the time until death attributed to NPC in the SEER database.

### Statistical Analysis

The continuous variable of age was transformed to categorical variable according to the median value. Categorical variables, including age, sex, race, tumor grade, pathology, chemotherapy, and secondary cancer, were analyzed by using the χ^2^ test or Fisher’s exact test. OS and CSS in the chemoradiotherapy and radiotherapy groups were calculated using Kaplan-Meier analysis with log-rank test statistics. Multivariable proportional hazards models adjusted for age, sex, race, tumor grade, pathology, chemotherapy, and secondary cancer were performed to assess independent prognostic factors. The results are reported as hazard ratios (HRs) with 95% confidence intervals (CIs).

Propensity score matching (PSM) was performed to reduce the influence of selection bias on the comparison of efficacy between the chemoradiotherapy and radiotherapy groups. A logistic regression model was established in which chemotherapy was considered the dependent variable in the process of calculating the propensity scores. One-to-one matching without replacement was completed using the nearest-neighbor match on the logit of the propensity score for confounding factors (derived from age, sex, race, tumor grade, pathology, and secondary cancer) with a caliper of 0.05.

Statistical analyses were performed using SPSS Statistics Version 26.0 software (IBM Co., Armonk, NY, USA) and R software (version 4.0.2). Two-tailed *P*  values <  0.05 were considered statistically significant.

## Results

### Patient Characteristics

**Figure 1:**
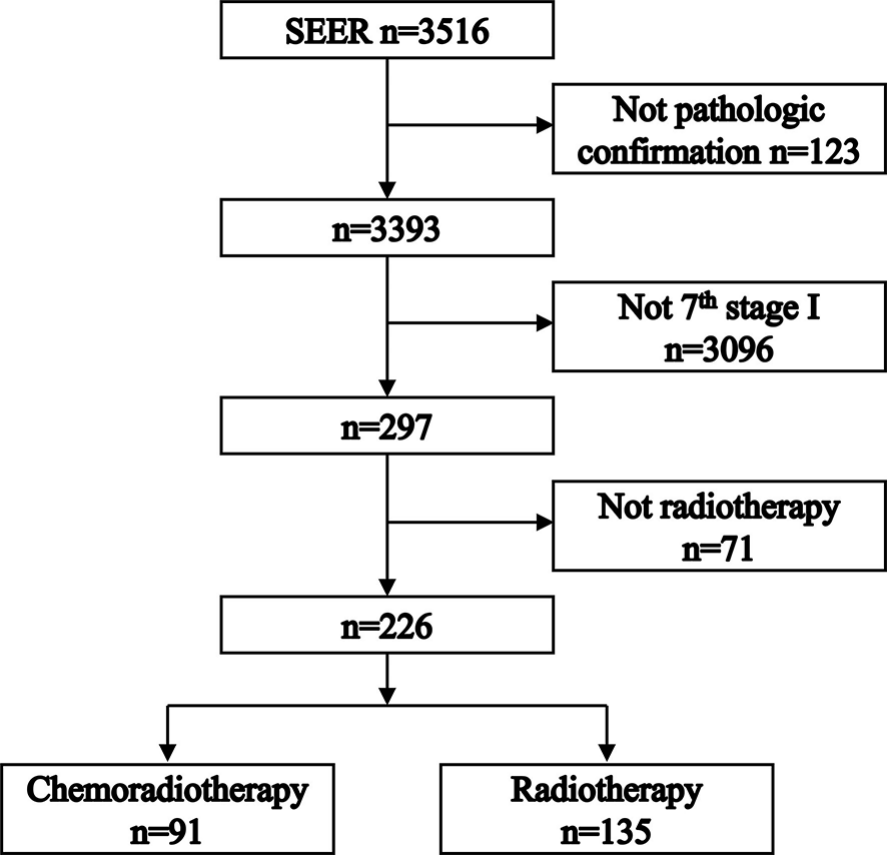
Flowchart depicting patient selection.

**Figure 1** shows the process of patient selection. A total of 226 patients were included. The chemoradiotherapy group included 91 (40.27%) patients. The radiotherapy group included 135 (59.73%) patients. **Table 1** shows the patient characteristics before and after PSM. In the PSM cohort, 86 patients receiving chemoradiotherapy and 86 patients receiving radiotherapy were matched. Patient characteristics were well balanced across all covariates after PSM (*P* > 0.05). The median follow-up times were 33 [interquartile range (IQR): 17–60] months for the chemoradiotherapy group and 43 (IQR: 22–63) months for the radiotherapy group, respectively.

**Table 1:** Patient characteristics.

	The unmatched cohort		*P*	The PSM cohort		*P*
	Radiotherapy (n=135)	Chemoradiotherapy (n = 91)		Radiotherapy (n = 70)	Chemoradiotherapy (n = 70)	
Age						
<60	63 (46.7%)	46 (50.5%)	0.567	35 (50.0%)	39 (55.7%)	0.498
≥60	72 (53.3%)	45 (49.5%)		35 (50.0%)	31 (44.3%)	
Sex						
Female	45 (33.3%)	19 (20.9%)	0.042	15 (21.4%)	18 (25.7%)	0.550
Male	90 (66.7%)	72 (79.1%)		55 (78.6%)	52 (74.3%)	
Race						
Asian	64 (47.4%)	29 (31.9%)	0.056	27 (38.6%)	27 (38.6%)	0.797
Black	10 (7.4%)	11 (12.1%)		4 (5.7%)	6 (8.6%)	
White	61 (45.2%)	51 (56.0%)		39 (55.7%)	37 (52.9%)	
Grade						
Unknown	39 (28.9%)	22 (24.2%)	0.523	17 (27.1%)	18 (25.7%)	0.938
I/II	16 (11.9%)	15 (16.5%)		10 (14.3%)	9 (12.9%)	
III/IV	80 (59.3%)	54 (59.3%)		41 (58.6%)	43 (61.4%)	
Pathology						
WHO I	28 (20.7%)	39 (31.9%)	0.259	11 (15.7%)	19 (27.1%)	0.424
WHO II	37 (27.4%)	24 (26.4%)		23 (32.9%)	20 (28.6%)	
WHO III	25 (18.5%)	12 (13.2%)		14 (20.0%)	11 (15.7%)	
Other	45 (33.3%)	26 (28.6%)		22 (31.4%)	20 (28.6%)	
Secondary cancer						
Yes	30 (22.2%)	31 (34.1%)	0.049	17 (24.3%)	18 (25.7%)	0.845
No	105 (77.8%)	60 (65.9%)		53 (75.7%)	52 (74.3%)	

WHO, World Health Organization; PSM, propensity score matching.

### Factors Associated With Chemotherapy Use

**Figure 2:**
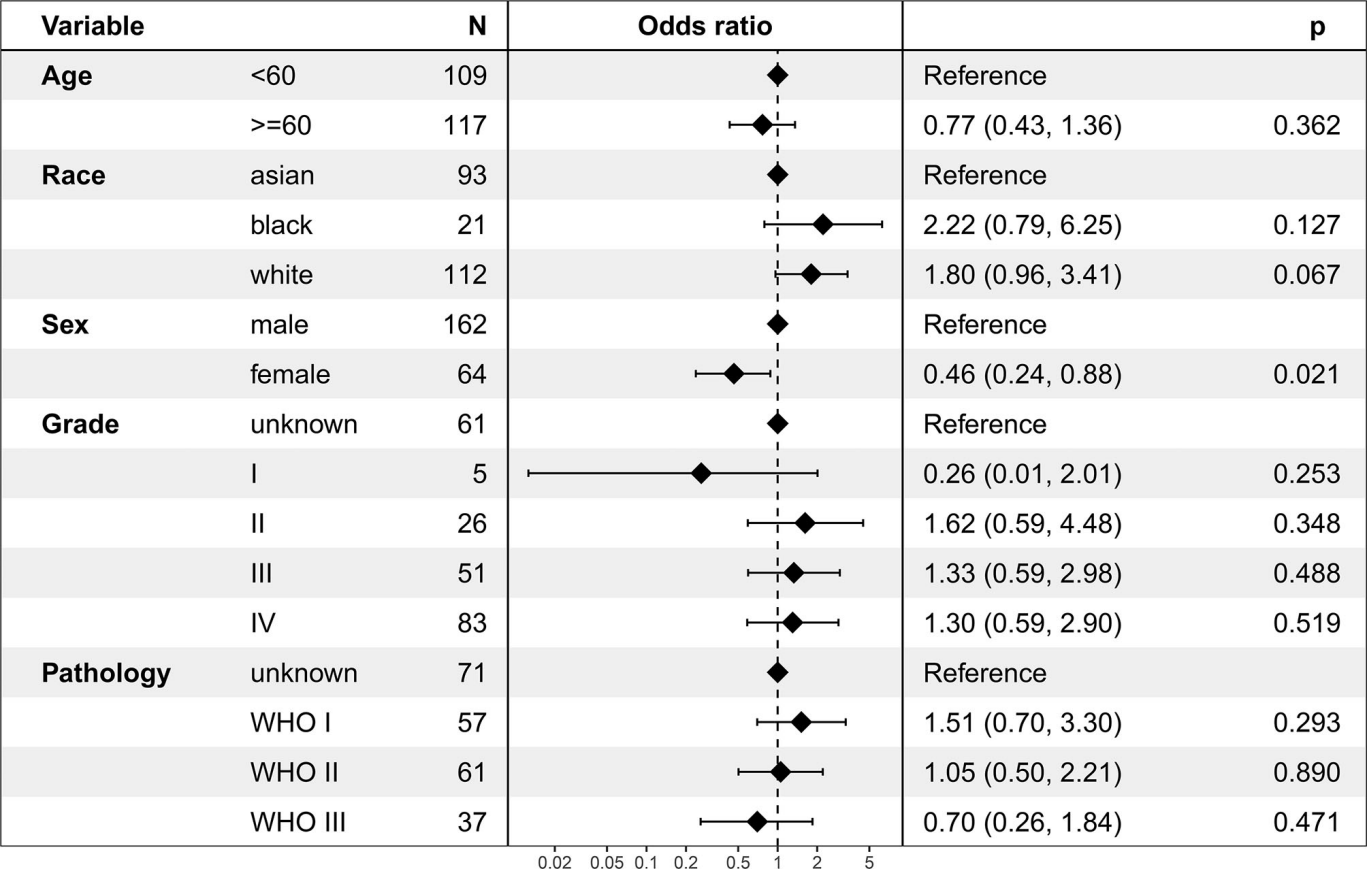
Logistic regression analysis for factors associated with chemotherapy use.

In the logistic regression analysis, chemotherapy use was associated with sex (**Figure 2**). Female [odds ratio (OR)=0.46, 95% CI: 0.24–.88; *P* = 0.021] was less likely to receive chemotherapy. Chemotherapy use was not associated with age, race, grade, or pathology.

### Survivals Before PSM

**Figure 3:**
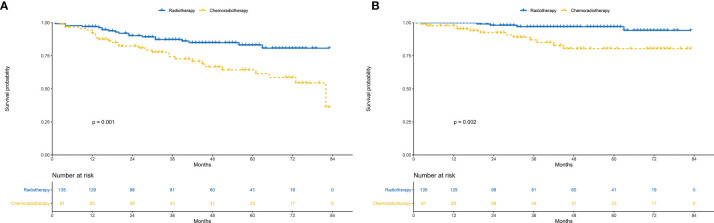
Survival between chemoradiotherapy and radiotherapy groups in the unmatched cohort. **(A)** Overall survival. **(B)** Cancer-specific survival.

Chemoradiotherapy group showed worse 3-year OS (74.31 *vs* 87.23%; *P* = 0.025) and 5-year OS (64.28 *vs* 83.12%; *P* = 0.001) than radiotherapy group (**Figure 3A**). Similarly, the 3-year CSS (87.01 *vs* 96.97%; *P* = 0.028) and 5-year CSS (80.39 *vs.* 96.97%; *P* = 0.002) of the chemoradiotherapy group were worse than that of the radiotherapy group (**Figure 3B**).

**Figure 4:**
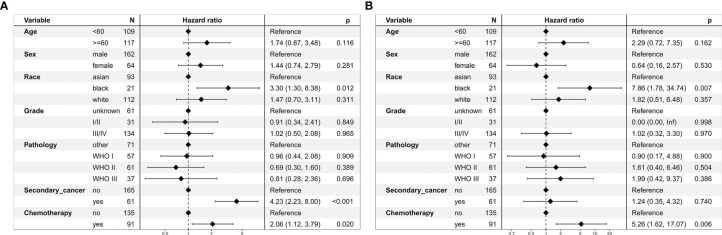
Multivariate regression analysis of prognostic factors for patients with stage I nasopharyngeal carcinoma in the unmatched cohort. **(A)** Overall survival. **(B)** Cancer-specific survival.

In the multivariate regression analysis, chemoradiotherapy was an independent risk prognostic factor for OS (HR = 2.06, 95% CI: 1.12–3.79; *P* = 0.020, **Figure 4A**) and CSS (HR = 5.26, 95% CI: 1.62–17.07; *P* = 0.006, **Figure 4B**).

### Survivals After PSM

**Figure 5:**
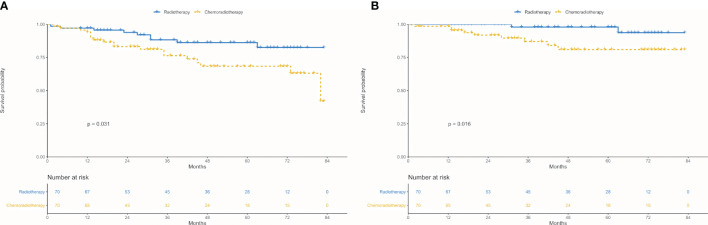
Survival between chemoradiotherapy and radiotherapy groups in the propensity-matched cohort. **(A)** Overall survival. **(B)** Cancer-specific survival.

In the matched cohort, chemoradiotherapy group revealed worse 5-year OS (63.10 *vs.* 82.49%; *P* = 0.031, **Figure 5A**) and CSS (80.95 *vs.* 93.70%; *P* = 0.016, **Figure 5B**) radiotherapy group.

**Figure 6:**
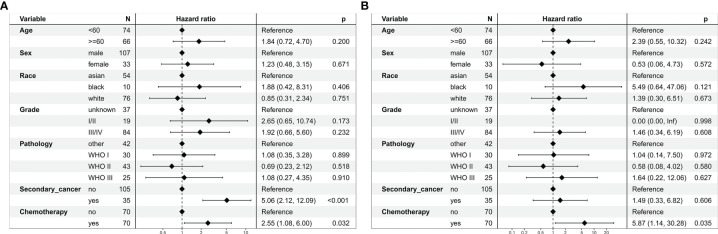
Multivariate regression analysis of prognostic factors for patients with stage I nasopharyngeal carcinoma in the propensity-matched cohort. **(A)** Overall survival. **(B)** Cancer-specific survival.

In the multivariate regression analysis, chemoradiotherapy was an independent risk prognostic factor for OS (HR = 2.55, 95% CI: 1.08–6.00; *P* = 0.032, **Figure 6A**) and CSS (HR = 5.87, 95% CI: 1.14–30.28; *P* = 0.035, **Figure 6B**).

## Discussion

This retrospective cohort study revealed that chemoradiotherapy was an independent risk prognostic factor for patients with stage I NPC. Addition of chemotherapy to stage I NPC decreased OS. It is recommended that patients with stage I NPC should not be treated with chemoradiotherapy.

It is difficult to conduct a randomized controlled trial due to the low incidence of stage I NPC (10). Thus, the efficacy of chemotherapy in survival of stage I NPC has not been well assessed. Several studies revealed that the 5-year OS was as high as 95.0% for stage I NPC patients receiving radiotherapy alone (5–7). Based on these studies, the National Comprehensive Cancer Network and the Chinese Society of Clinical Oncology are recommended radiotherapy alone for this subgroup patients. However, biases inherently existed in these retrospective studies. On the other hand, stage I NPC patients did not receive chemotherapy in these studies (5–7).

This raised the question of whether chemoradiotherapy could improve survival of this subgroup patients. In clinical practice, clinicians might suggest patients to receive chemotherapy for a potential better prognosis. A previous study included 396 stage I NPC patients of the National Cancer Data Base revealed that 147 (37.12%) patients underwent chemoradiotherapy (11). Similarly, our study based on the SEER database showed that 40.27% patients received chemoradiotherapy. Thus, it is important to identify the efficacy of chemotherapy in survival of this subgroup patient.

Verma et al. (11) reported that the 5-year OS in stage I NPC patients receiving radiotherapy alone *versus* chemoradiotherapy were 77 and 75% (*P* = 0.428). Receipt of chemotherapy did not independently predict for better OS (HR = 1.195, 95% CI: 0.755–1.893; *P* = 0.447). In contrast, our study revealed that chemotherapy was an independent risk prognostic factor for OS. The possible reasons for the difference of the two studies were as follows: (1) Verma et al. (11) assessed patients from the National Cancer Data Base between 2004 and 2013. Among the 396 patients, only 132 (33.33%) patients were diagnosed after 2010. Chemoradiotherapy group included 41 patients. The sample size was too small to perform statistical analysis. (2) Patients were mainly tested by computed tomography before 2010. Several stage II patients might mis-stage as stage I NPC in the study of Verma et al. (11). Chemotherapy might improve survival of stage II patients.

However, treatment outcomes of local-regional failure and distant failure were crucial to assess the efficacy of chemotherapy on stage I NPC. Unfortunately, these data were not recorded in the SEER database and the National Cancer Data Base. Moreover, salvage treatments for local-regional recurrences and distant metastasis were not available. The salvage therapies including re-irradiation, surgery, or systemic chemotherapy could influence OS. Thus, the different results between the two studies were not identified.

Our study suggested that chemotherapy decreased OS of patients with stage I NPC. Firstly, chemoradiotherapy increased toxicity reactions compared to radiotherapy (12, 13). Severe hematological adverse events might be life-threatening. Moreover, long-term survivors of stage I patients would have problems with eating, swallowing, hearing, and psychological and functional problems (14). These toxicity reactions might lead to poor survival in the chemoradiotherapy group. Secondly, the proportion of secondary cancer in chemoradiotherapy group was higher than that of radiotherapy group (*P* = 0.049). The multivariate regression analysis revealed that secondary cancer was an independent risk prognostic factor for OS (HR = 2.06, 95% CI: 1.12–3.79; *P* = 0.020), which would decrease OS of the chemoradiotherapy group.

On the other hand, it was an unexpected finding that chemoradiotherapy decreased CSS of stage I NPC in our study. We considered that the result might be a statistical bias. The potential interpretations were the following: (1) Before PSM, 4 patients in the radiotherapy group and 12 patients in the chemoradiotherapy group attributed to NPC death. The sample size of endpoint events was not sufficient to compare the differences between the two groups using Kaplan-Meier analysis with log-rank test statistics. The statistical analysis might provide a false positive result. (2) The 95% confidence interval ranged from 1.62 to 17.07 before PSM and from 1.14 to 30.28 after PSM. The wide confidence interval indicated an unstable statistical result. Thus, longer follow-up time and larger sample size of endpoint events are needed to verify the result.

Limitations of our study should be noted. First, a total of 91 patients received chemoradiotherapy. The small sample size of patients who received chemoradiotherapy might have been insufficient for statistical analysis. This would possibly significantly reduce the statistical power of the analysis. Second, data regarding the performance status, Epstein-Barr virus DNA (15), chemotherapy agents, irradiation dose, or the duration of therapy were not available due to limitations of the SEER database. These confounding factors could affect survival. Thus, further prospective cohort studies are needed to identify the efficacy of chemotherapy in survival of stage I NPC.

## Conclusion

In conclusion, chemotherapy decreased OS of patients with stage I NPC. Radiotherapy alone is recommended for stage I NPC patients.

## Data Availability Statement

The original contributions presented in the study are included in the article/**Supplementary Material**. Further inquiries can be directed to the corresponding author.

## Author Contributions

X-BP contributed to the conception of the study. J-LM, S-TH, and Y-MJ performed the data analyses, contributed to manuscript preparation, and helped to perform the analysis with constructive discussions. All authors contributed to the article and approved the submitted version.

## Funding

This study was supported by the grant of Department of Education of Guangxi Zhuang Autonomous Region (No. KY2016LX029), the grant of Guangxi Medical University (No. GXMUYSF201521), the Research and Development Project of Guangxi (No. 1598012-22 and No. AB18221007).

## Conflict of Interest

The authors declare that the research was conducted in the absence of any commercial or financial relationships that could be construed as a potential conflict of interest.

## Publisher’s Note

All claims expressed in this article are solely those of the authors and do not necessarily represent those of their affiliated organizations, or those of the publisher, the editors and the reviewers. Any product that may be evaluated in this article, or claim that may be made by its manufacturer, is not guaranteed or endorsed by the publisher.
